# DB-MLP: A Lightweight Dual-Branch MLP for Road Roughness Classification Using Vehicle Sprung Mass Acceleration

**DOI:** 10.3390/s26030990

**Published:** 2026-02-03

**Authors:** Defu Chen, Mingye Li, Guojun Chen, Junyu He, Xiaoai Lu

**Affiliations:** 1 College of Information Engineering, Zhejiang University of Technology, Hangzhou 310023, China; defuchen@zjut.edu.cn (D.C.); 221125030359@zjut.edu.cn (J.H.); 2 Wanxiang Precision Industry Co., Ltd., Hangzhou 311202, China; 3 Wanxiang Qianchao Co., Ltd., Hangzhou 311215, China

**Keywords:** road roughness classification, deep learning, DB-MLP, vehicle dynamics simulation, suspension control, lightweight model

## Abstract

Accurate identification of road roughness is pivotal for optimizing vehicle suspension control and enhancing passenger comfort. However, existing data-driven methods often struggle to balance classification accuracy with the strict computational constraints of real-time onboard monitoring. To address this challenge, this paper proposes a lightweight and robust road roughness classification framework utilizing a single sprung mass accelerometer. First, to overcome the scarcity of labeled real-world data and the limitations of linear models, a high-fidelity co-simulation platform combining CarSim and Simulink is established. This platform generates physically consistent vibration datasets covering ISO A–F roughness levels, effectively capturing nonlinear suspension dynamics. Second, we introduce DB-MLP, a novel Dual-Branch Multi-Layer Perceptron architecture. In contrast to computationally intensive Transformer or RNN-based models, DB-MLP employs a dual-branch strategy with multi-resolution temporal projection to efficiently capture multi-scale dependencies, and integrates dual-domain (time and position-wise) feature transformation blocks for robust feature extraction. Experimental results demonstrate that DB-MLP achieves a superior accuracy of 98.5% with only 0.58 million parameters. Compared to leading baselines such as TimeMixer and InceptionTime, our model reduces inference latency by approximately 20 times (0.007 ms/sample) while maintaining competitive performance on the specific road classification task. This study provides a cost-effective, high-precision solution suitable for real-time deployment on embedded vehicle systems.

## 1. Introduction

Road roughness is a critical indicator for assessing pavement quality, as it directly impacts vehicle dynamics and passenger ride comfort. Irregularities in the road surface induce vertical vibrations that are transmitted to the vehicle body, which can deteriorate driving stability and increase passenger fatigue [1]. Beyond comfort, advanced AI-powered detection systems have been increasingly recognized for their potential to enhance road safety and provide life protection by identifying hazardous damage early [2]. Therefore, accurate identification and classification of road surface conditions are vital. The International Standards Organization (ISO) classifies road roughness into classes A through F based on Power Spectral Density (PSD), a standard widely adopted in simulation and engineering practices [3].

Traditional methods for measuring road roughness are generally categorized into contact-based and non-contact-based approaches. Contact-based methods, such as profilers, offer high accuracy but are limited in scalability and high-speed applicability [4]. In the realm of non-contact approaches, recent studies have successfully employed Convolutional Neural Networks (CNNs) to classify road environments using visual data [5] and assess terrain roughness based on ISO standards [6]. While these visual and sensor-based machine learning techniques show promise [7], traditional feature engineering relying on handcrafted statistical indicators, such as RMS and kurtosis, often fails to capture the complex nonlinear mapping between road excitation and vehicle response [8].

In accelerometer-based approaches, sensor placement is a pivotal design choice. Early studies typically placed sensors at the wheel center (unsprung mass) to classify roughness conditions using methods such as sliding time windows [9,10]. While this configuration captures raw road profiles directly, it faces severe engineering challenges due to the harsh working environment. Furthermore, raw wheel-end signals contain excessive high-frequency noise. In contrast, reusing existing sprung mass accelerometers offers a robust alternative. Not only is this approach cost-effective by leveraging standard onboard sensors or low-cost MEMS units, a direction validated by recent monitoring studies [11], but the suspension system also acts as a natural filter for irrelevant high-frequency noise, aligning better with ride comfort assessment [1].

Given the costs associated with real-world data collection, simulation has become a pivotal approach for generating training datasets. However, widely utilized linear quarter-car models often oversimplify suspension components. To address these deficiencies and capture nonlinear dynamic characteristics, recent research has increasingly relied on joint simulation environments and real-vehicle tests to validate speed-adaptive classification frameworks [8]. This study establishes a high-fidelity full-vehicle model to generate realistic vibration feature signals essential for training robust classifiers.

To effectively process these complex signals and overcome the limitations of manual feature extraction, Deep Learning (DL) models such as CNNs [12], RNNs [13], and Transformers have been explored. Specifically, Long Short-Term Memory (LSTM) networks [14] were introduced to capture temporal dependencies, while Transformer architectures [15] and their time-series variants like Informer [16] have shown promise in modeling long-range correlations. However, existing architectures face limitations in vehicular edge computing scenarios. LSTM suffer from serial execution bottlenecks, preventing parallel computation and limiting inference speed on embedded chips. Transformers, while capable of modeling long-range dependencies, incur heavy computational costs due to self-attention mechanisms. Moreover, they treat continuous physical signals as discrete tokens, which may result in the loss of phase continuity information.

To overcome these challenges in both hardware deployment and algorithm efficiency, the main contributions of this paper are as follows:

- We propose a road roughness classification framework utilizing a single sprung mass accelerometer. The framework is primarily validated on high-fidelity synthetic data generated from a joint CarSim–Simulink platform covering ISO A–F roughness levels. Furthermore, to verify the robustness and generalization capability of our model under realistic engineering constraints, we conduct comprehensive stress tests across varying environmental noise levels, vehicle mass configurations, and driving speeds, confirming its reliability for real-world deployment.
- We conduct a comprehensive comparison of various deep learning models, systematically analyzing the trade-offs between classification accuracy and computational efficiency in the context of vehicular edge computing.
- We propose DB-MLP, a novel dual-branch architecture based purely on MLPs. Unlike Transformers, it eliminates complex tokenization and heavy attention mechanisms; unlike LSTMs, it supports fully parallel inference. The proposed model achieves superior accuracy with minimal parameters, making it an ideal candidate for real-time deployment.

## 2. Related Work

### 2.1. High-Fidelity Data Generation Pipeline

Reliable road roughness classification hinges on high-quality and physically consistent training data. Due to the high cost and logistical challenges of collecting labeled real-world datasets, simulation has become a cost-effective and reliable data source [7]. Early research often relied on simplified linear quarter-car models implemented in MATLAB (version R2024a)/Simulink [9]. While computationally efficient, these linear models fundamentally fail to capture the complex non-linear dynamics inherent in real suspension systems. Specifically, Wu et al. [17] demonstrated that suspension hysteresis, arising from leaf spring friction and non-linear damping, significantly alters vehicle vibration performance and affects amplitude response in the low-frequency range. Similarly, studies on pneumatic suspensions by Ho et al. [18] highlight that traditional linear models struggle to balance comfort and robustness under complex operating conditions due to strong non-linearities and parameter uncertainties.

To bridge the gap between simulation and reality, this study adopts a rigorous high-fidelity co-simulation framework. Prior research has extensively validated the reliability of such platforms for complex vehicle dynamics. For instance, Jiang and Qiu [19] utilized a joint CarSim-Simulink platform to model Vehicle Stability Control systems and successfully handled non-linear vehicle attitudes through adaptive fuzzy PID control. Furthermore, Liu et al. [20] verified the high fidelity of the platform by reconstructing powertrain systems for in-wheel motor electric vehicles, achieving seamless cross-platform coupling that preserved dynamic accuracy.

In this work, we employ this proven pipeline. Random road profiles are first generated in MATLAB/Simulink strictly adhering to the ISO 8608 [21] standard based on Power Spectral Density (PSD). These profiles serve as excitation inputs for CarSim, which accurately models specific production vehicle parameters. This two-stage process ensures that the generated sprung-mass acceleration signals preserve both the stochastic nature of road inputs and the non-linear response characteristics of the vehicle, providing a solid physical foundation for training deep learning models.

### 2.2. Deep Learning Models for Time-Series Classification

The task of classifying road roughness from vibration signals requires balancing feature extraction capability with computational efficiency. The field has evolved through several stages, each with distinct limitations for this specific application:

- RNNs and CNNs: Recurrent Neural Networks, particularly LSTMs [14], were early favorites for capturing temporal dependencies. For instance, Liang et al. [10] proposed an end-to-end LSTM framework that directly processes time-series acceleration signals to identify road roughness, demonstrating high accuracy and strong robustness across varying vehicle speeds and suspension parameters. However, RNNs suffer from serial processing bottlenecks that hinder hardware parallelization and increase latency. Convolutional Neural Networks offer faster processing. Notably, Sabapathy and Biswas [22] utilized CNNs to process raw OBD-II acceleration and speed data, achieving significantly better identification of poor road conditions compared to traditional methods like SVMs. Yet, standard CNNs are often limited by local receptive fields and struggle to capture the global signal trends necessary for robust classification.
- Transformers: To address global dependency modeling, Transformer-based architectures [15] and variants like Informer [16] utilize self-attention mechanisms. While they achieve high accuracy by modeling long-range correlations, their heavy computational burden and high memory footprint make them impractical for deployment on resource-constrained vehicular edge devices where real-time latency is critical.
- Existing MLP-Based Architectures: Recently, pure MLP architectures such as LightTS [23] and TimeMixer [24] have gained popularity due to their high inference speed. However, most of these models are designed for generic time-series forecasting rather than classification. They tend to prioritize capturing smooth long-term trends and often sacrifice the ability to distinguish the subtle, high-frequency vibration patterns inherent in similar road roughness classes. In short, while they solve the efficiency problem, they often lack the precision and feature expressiveness required for effective suspension control systems. While established research using convolutional or recurrent structures has investigated model stability under varying driving conditions, recent lightweight architectures based on multilayer perceptrons often prioritize predictive performance on standard benchmarks. Consequently, there remains a need for a systematic evaluation of these efficient models against critical engineering constraints, such as sensor noise and fluctuations in vehicle mass.

### 2.3. The Proposed DB-MLP Approach

To address the aforementioned challenges, this work proposes the Dual-Branch MLP (DB-MLP). The primary objective is to bridge the gap between the high accuracy of complex models and the computational efficiency of lightweight MLPs. Unlike conventional MLP architectures that process temporal features at a single resolution, DB-MLP introduces a specialized dual-branch strategy to explicitly capture multi-scale dependencies. This mechanism effectively integrates high-frequency vibration textures with low-frequency global motion trends. Consequently, the proposed design attains accuracy levels comparable to resource-intensive architectures while maintaining a compact parameter footprint suitable for real-time embedded deployment.

## 3. Methodology

### 3.1. Overall Framework

**Figure 1:**
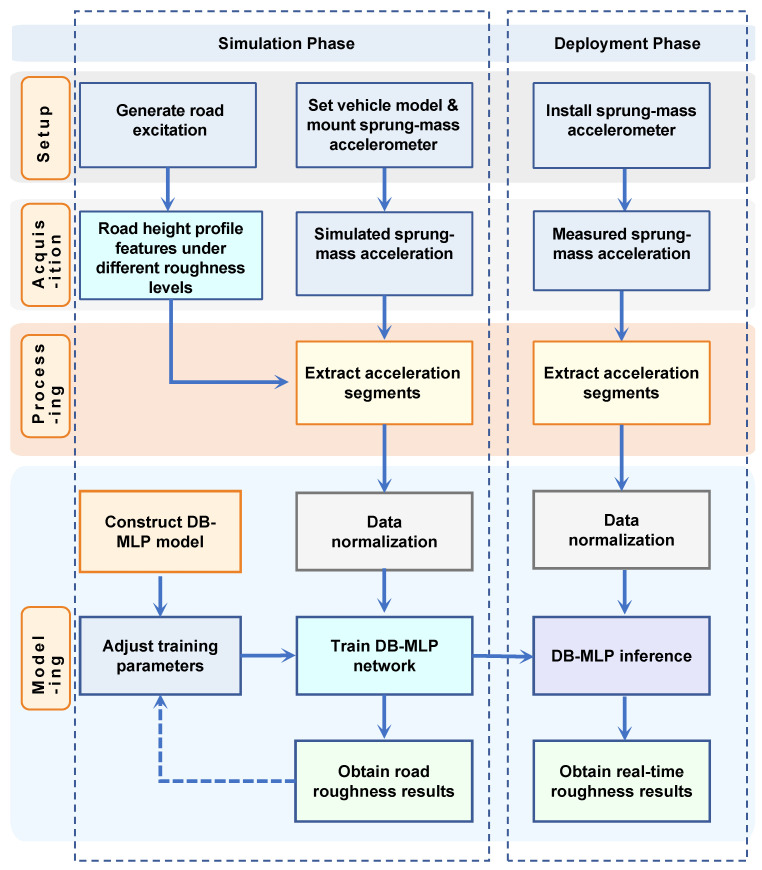
Overall framework for end-to-end road roughness classification.

The proposed framework performs integrated road roughness classification based on simulated vibration data, combining physical modeling, signal preprocessing, and a neural network classifier (Figure 1).

Road surface profiles corresponding to ISO 8608 roughness levels (A–F) are generated and used as excitation inputs in a full-vehicle dynamics simulation. The resulting vertical acceleration of the sprung mass is recorded through a virtual accelerometer mounted at the vehicle’s center of gravity. The acceleration signals are sampled at 1000 Hz, segmented into 1 s windows, normalized, and labeled according to the corresponding road class, forming a physically consistent training dataset with representative vibration characteristics.

The classification model is implemented using the proposed DB-MLP architecture, a lightweight multi-branch MLP framework specifically designed for vibration-based classification. DB-MLP extracts multi-scale temporal patterns through nonlinear temporal projection and a generic classification head, without relying on decomposition or attention mechanisms.

Finally, the trained DB-MLP network outputs one of six ISO-defined roughness levels. This framework integrates physical simulation and data-driven modeling to achieve efficient and accurate road surface classification using a single accelerometer configuration.

### 3.2. Simulation-Based Data Generation and Preprocessing

To obtain labeled, high-quality sensor data for surface classification, we simulate road-induced vehicle responses based on the ISO 8608 standard. This standard defines road roughness as a stochastic process characterized by its Power Spectral Density (PSD). Each roughness class (A–F) corresponds to a different PSD level.

#### 3.2.1. Road Surface Roughness Model

The road surface elevation is modeled as a zero-mean stationary Gaussian process. Its spatial frequency characteristics are described by the PSD function:

(1) $$G_{q}\left(n\right)=G_{q}\left(n_{0}\right){\left(\frac{n}{n_{0}}\right)}^{-W}$$

Here, *n* is the spatial frequency $\left[m^{-1}\right]$, and $n_{0}=0.1\;m^{-1}$ is the reference frequency. $G_{q}\left(n_{0}\right)$ denotes the road roughness coefficient, which varies with ISO-defined classes (A–F). W=2 is the typical frequency index that controls how roughness energy distributes over frequency. This model allows generating synthetic road profiles with specific statistical properties.

To analyze road profiles in the temporal domain, the PSD can be transformed as:

(2) $$G_{q}\left(\omega \right)={\left(2\pi \right)}^{2}n_{0}^{2}G_{q}\left(n_{0}\right)\frac{u}{\omega_{0}^{2}+\omega^{2}}$$

where ω is the angular frequency, *u* is vehicle speed, and $\omega_{0}$ is the cutoff frequency.

This temporal representation enables simulation via linear shaping filters.

The corresponding shaping filter transfer function is:

(3) $$H\left(j\omega \right)=\frac{2\pi n_{0}\sqrt{G_{q}\left(n_{0}\right)u}}{\omega_{0}+j\omega }$$

The road roughness evolution can also be expressed as a stochastic differential equation:

(4) $$\dot{q}\left(t\right)=-2\pi n_{1}uq\left(t\right)+2\pi n_{0}\sqrt{G_{q}\left(n_{0}\right)u}\;w\left(t\right)$$

where q(t) represents the road surface roughness displacement [m];

$n_{1}$ = $0.01\;m^{-1}$ is the lower cutoff spatial frequency and w(t) is a zero-mean white noise signal with a unit power spectral density.

Equation (4) characterizes the road surface as a first-order stochastic process driven by filtered white noise, effectively capturing the statistical properties of road roughness for simulation and vibration analysis.

#### 3.2.2. Simulink-Based Profile Generation

The road profile generation model was implemented within the MATLAB/Simulink environment, grounded in the mathematical formulation of Equation (4). A standard Gaussian white noise module served as the stochastic source w(t), configured with sufficient sampling resolution to accurately capture high-frequency road features. This noise signal acts as the excitation input to a continuous transfer function block, which shapes the spectral content to produce the time-domain road elevation q(t).

To simulate a comprehensive range of driving scenarios, independent road profiles corresponding to ISO 8608 roughness classes A through F were generated. The roughness coefficient $G_{q}\left(n_{0}\right)$ for each class is constrained within the lower and upper limits specified in Table 1, with the geometric mean serving as the reference baseline. The vehicle speed *u* within the model was fixed at 10 m/s to ensure kinematic consistency with the subsequent full-vehicle dynamics simulation.

**Figure 2:**
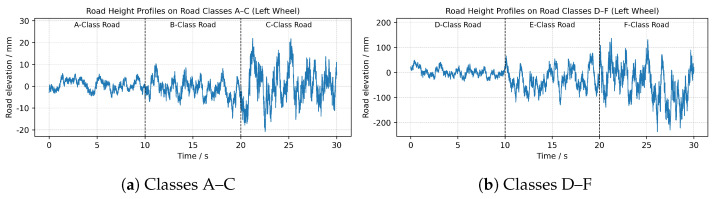
Time-domain road roughness signals generated. (**a**) Profiles for ISO classes A–C; (**b**) Profiles for ISO classes D–F.

The resulting time-domain roughness signals at a speed of 10 m/s are illustrated in Figure 2. Figure 2a displays the relatively smooth profiles for classes A and C, while Figure 2b depicts the significantly higher amplitudes associated with the rougher conditions of classes D and F.

### 3.3. Road Profile Generation and Validation

**Figure 3:**
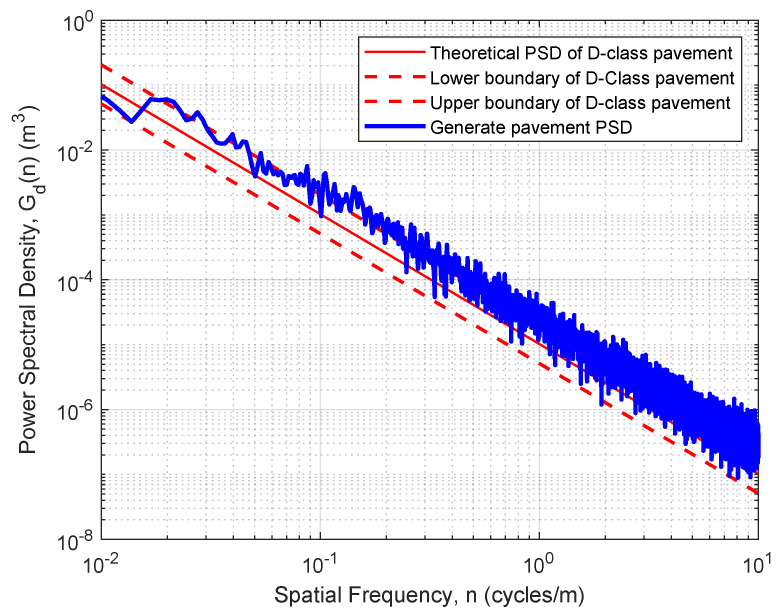
PSD comparison between the generated road profile and the ISO 8608 D-class region.

Road roughness signals are generated in Simulink by filtering white noise to obtain road surface profiles that follow the target power spectral density (PSD). Each simulated profile is evaluated by comparing its PSD with the ISO 8608 reference spectrum. The lower and upper tolerance limits are defined as one-half and twice the standard PSD, respectively, as summarized in Table 1. As shown in Figure 3, the generated profile falls within the D-class region, confirming the accuracy of the road roughness modeling. The validated elevation data are then exported via the To Workspace block and imported into CarSim for full-vehicle simulation.

### 3.4. Vehicle Response Simulation and Labeling

To generate a high-fidelity labeled dataset, a production-level mid-size SUV model was established within the CarSim environment. The vehicle simulated driving over generated ISO 8608 road profiles (Classes A–F) at a constant speed of 10 m/s. The suspension system employed a double-wishbone (SLA) configuration at the front and a Hotchkiss solid axle at the rear.

Crucially, to replicate realistic driving conditions and induce authentic vehicle roll dynamics, distinct road profiles were generated for the left and right wheels. While both tracks correspond to the same ISO roughness class, they were synthesized using independent random seeds and slightly varying roughness coefficients ($G_{q}\left(n_{0}\right)$) within the standardized limits. This spatial asymmetry ensures that the model captures complex vehicle body motions beyond simple vertical heave.

The primary feature for classification is the sprung-mass vertical acceleration, recorded by a virtual sensor at the vehicle’s center of gravity with a sampling rate of 1000 Hz. Physically, the vehicle suspension acts as a mechanical low-pass filter, effectively attenuating high-frequency disturbances from tire-road interactions and engine vibrations while preserving the low-to-mid frequency components that characterize road roughness. The continuous acceleration signals were then segmented into 1-s windows and labeled according to the corresponding road roughness class. The key physical parameters of the vehicle model are detailed in Table 2.

**Figure 4:**
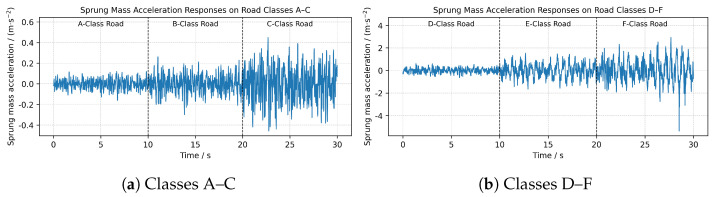
Simulated sprung mass vertical acceleration responses. (**a**) Classes A–C; (**b**) Classes D–F.

The simulated acceleration responses are illustrated in Figure 4. While a global trend of increasing signal amplitude is observable from Class A to F, amplitude alone is often insufficient for precise classification due to the significant overlap between adjacent classes.

- Classes A–C (Figure 4a): Exhibit continuous, stationary random vibrations with relatively low amplitudes.
- Classes D–F (Figure 4b): Induce significantly higher vertical excitations. Notably, these rougher roads trigger nonlinear suspension dynamics, characterized by transient high-energy impacts and distinct decay patterns.

These temporal and spectral variations, rather than simple energy thresholds, provide the critical discriminative information for the proposed model.

### 3.5. DB-MLP Architecture

#### 3.5.1. Motivation and Background

Sprung-mass acceleration signals generated by road excitation exhibit strong non-stationarity, broadband frequency content, and short-duration high-energy transients. These characteristics differ from the long-term temporal dependencies commonly targeted by forecasting-oriented architectures such as RNNs and Transformers. In the present road-surface classification setting, the sequence length is moderate (1 s, 1000 samples) and the discriminative information is mainly encoded in local vibration patterns rather than very long-range dependencies.

Transformer-based models and other deep attention architectures have demonstrated strong performance on generic time-series benchmarks and can also achieve competitive accuracy in our setting. However, they typically require substantially more parameters, higher computational cost, and increased inference latency, which limits their suitability for real-time, embedded deployment with low-cost onboard hardware.

Conversely, recent linear time-series models show that simple temporal projections can be surprisingly effective for forecasting, but their purely linear mappings lack the nonlinear capacity needed to fully separate road-induced vibration patterns, especially in high-frequency bands.

**Figure 5:**
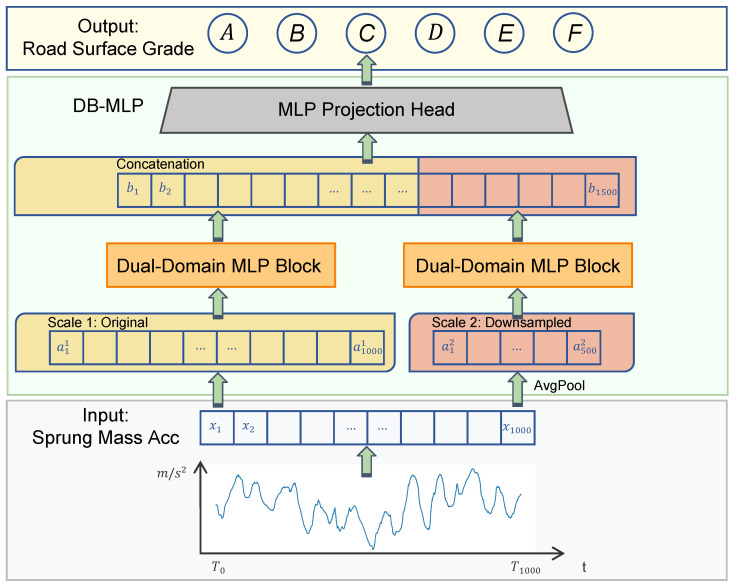
Schematic of the proposed DB-MLP architecture.

To balance accuracy and efficiency, we propose a lightweight DB-MLP (Dual-Branch MLP) architecture. The design philosophy is to use nonlinear MLP projectors to extract discriminative vibration features, while a multi-branch structure captures multiple temporal resolutions without resorting to complex attention mechanisms. This yields a compact model that aligns well with the characteristics of high-frequency sprung-mass acceleration and is better suited to real-time deployment on resource-constrained platforms. The overall architecture is illustrated in Figure 5, and the detailed specifications are listed in Table 3.

#### 3.5.2. Dual-Domain MLP Block

The core processing unit of DB-MLP is the Dual-Domain MLP Block. To efficiently model the dynamic characteristics inherent in vibration signals, this block employs a decoupled design that sequentially applies temporal mixing and position-wise feature transformation. This separation significantly reduces the parameter complexity compared to standard dense layers.

Time Mixing

First, a time-mixing MLP captures global temporal dependencies. We reinterpret the time dimension $L_{k}$ as the feature axis, projecting it into a latent space of dimension $d_{model}$ and then restoring it. Omitting bias terms for brevity, the operation is defined as:

(5) $$T^{\left(k\right)}=W_{t2}\cdot \phi \;\left(W_{t1}X^{\left(k\right)}\right),$$

where ϕ denotes the GELU activation. Crucially, this bottleneck structure minimizes redundancy: $W_{t1}\in R^{d_{model}\times L_{k}}$ compresses the temporal sequence, and $W_{t2}\in R^{L_{k}\times d_{model}}$ reconstructs it. The total parameters for this module scale linearly with $L_{k}\cdot d_{model}$, avoiding the quadratic complexity of full attention mechanisms.

Position-wise Feature Transformation

Subsequent to the temporal projection, the second stage concentrates on the local feature domain. Whereas conventional Transformer architectures typically utilize a Position-wise Feed-Forward Network to project features into a high-dimensional latent space via an expansion-reduction mechanism, our analysis indicates that such dimensional expansion introduces unnecessary redundancy and heightens the risk of overfitting for univariate inputs possessing a single channel.

To mitigate this issue, we substitute the standard FFN with a streamlined Position-wise Feature Transformation block. This module is instantiated as a learnable Adaptive Scaling Layer that performs a non-linear transformation independently at each time step to calibrate the signal amplitude:

(6) $$H^{\left(k\right)}=W_{c2}\cdot \psi \;\left(W_{c1}T^{\left(k\right)}\right).$$

In this formulation, $W_{c1}$ and $W_{c2}$ denote learnable scalar weights within $R^{1\times 1}$, and ψ represents the GELU activation function. By eschewing the extensive parameterization characteristic of standard FFNs, this module functions as a critical dynamic gain controller. It operates orthogonally to the Time Mixing layer by selectively amplifying significant resonance patterns or attenuating noise based on signal intensity, thereby maximizing parameter efficiency for resource-constrained edge devices.

#### 3.5.3. Feature Fusion and Classification

The final stage integrates the learned representations from all temporal branches to form a holistic description of the road surface. Since each branch outputs a univariate feature sequence $H^{\left(k\right)}\in R^{L_{k}\times 1}$, we flatten and concatenate them to construct a unified global feature vector:

(7) $$H_{fused}=\mathrm{Concat}\;\left(H^{\left(0\right)},H^{\left(1\right)},\ldots ,H^{\left(D\right)}\right).$$

The dimensionality of this fused vector is the sum of temporal lengths across all scales, $P=\sum_{k}L_{k}$. In our specific configuration ($L_{0}=1000,L_{1}=500$), this results in a comprehensive feature vector $H_{fused}\in R^{1500}$, preserving both fine-grained high-frequency details and coarse-grained low-frequency trends.

Finally, a lightweight MLP projection head maps this high-dimensional representation to the semantic label space. Consistent with previous blocks, we simplify the notation by omitting bias terms:

(8) $$o=W_{f2}\cdot \phi \;\left(W_{f1}H_{fused}\right),$$

where $W_{f1}\in R^{d_{model}\times P}$ projects the fused features into the latent hidden space, and $W_{f2}\in R^{K\times d_{model}}$ generates the final logits for the K=6 road surface classes.

#### 3.5.4. Model Complexity and Efficiency

To demonstrate the lightweight nature of DB-MLP, we analyze its parameter complexity (P) and computational complexity (O). Let $L_{total}=\sum_{k}L_{k}$ denote the cumulative sequence length across all branches. The parameter distribution is analyzed as follows:

- Time Mixing Layers: As the primary feature extractors, these layers perform global projection across time. Their parameter count scales linearly with the sequence length: $P_{time}\approx 2\cdot L_{total}\cdot d_{model}$.
- Classification Head: The first projection layer of the MLP head maps the fused temporal vector to the hidden space, contributing $P_{head}\approx L_{total}\cdot d_{model}$.
- Position-wise Transformation: Due to the univariate input design (C=1), the adaptive scaling layers contribute a constant and negligible amount of parameters relative to the sequence length.

Aggregating these components, the total parameter count is dominated by the linear terms:

(9) $$P_{total}\approx P_{time}+P_{head}\approx 3\cdot L_{total}\cdot d_{model}.$$

For our experimental configuration ($L_{total}=1500,d_{model}=128$), the model contains approximately 0.58 million parameters.

In terms of computational complexity, DB-MLP achieves linear complexity with respect to the sequence length, i.e., $O(L_{total}\cdot d_{model})$. This represents a substantial efficiency advantage over Transformer-based architectures, whose self-attention mechanism incurs a quadratic complexity of $O(L_{total}^{2})$.

#### 3.5.5. Design Summary

In summary, the proposed DB-MLP architecture integrates three distinguishing characteristics tailored for vibration signal analysis:

- Physics-Aware Multi-Resolution Modeling: By employing a dual-branch structure, the model simultaneously captures high-frequency transient impacts and low-frequency suspension trends, addressing the broadband nature of road excitations.
- Linear-Complexity Feature Extraction: The decoupled Time-Mixing and Position-wise Transformation design avoids the quadratic bottleneck of self-attention mechanisms, ensuring O(L) computational efficiency.
- Holistic Representation Integration: The model fuses global temporal context across scales into a unified vector, creating a highly separable latent space that simplifies the downstream classification task.

Collectively, these design choices establish DB-MLP as a superior trade-off solution. By delivering highly competitive accuracy with a minimal parameter footprint, the architecture demonstrates strong theoretical feasibility for future deployment on resource-constrained automotive control units, see Table 4.

### 3.6. Training Strategy

The training process involves a physics-preserving preprocessing step followed by a supervised classification objective.

#### 3.6.1. Physics-Aware Normalization

Standard time-series normalization often employs instance-wise scaling, such as normalizing each sample independently to zero mean and unit variance. However, in road surface classification, the absolute amplitude of the acceleration signal carries critical physical information. Specifically, rough roads like Class E or F are characterized by high-energy impacts where the suspension often hits its limit stops (bump stops), generating peak values significantly higher than those of smooth roads.

If instance-wise normalization were applied, the low-amplitude noise of Class A roads would be rescaled to the same numerical range as the high-impact signals of Class F, thereby destroying the relative energy features between classes. To preserve the physical consistency of vibration magnitudes, we adopt a Global Normalization strategy. The normalized input $\hat{X}$ is computed as:

(10) $$\hat{X}=\frac{X-\mu_{\mathrm{train}}}{\sigma_{\mathrm{train}}},$$

where $\mu_{\mathrm{train}}$ and $\sigma_{\mathrm{train}}$ are the global mean and standard deviation calculated over the entire training dataset. This ensures that the distinct signal signatures of suspension limit impacts are retained in the input feature space.

#### 3.6.2. Optimization Objective

We formulate the road surface recognition task as a multi-class classification problem. Given the normalized input sequence $\hat{X}\in R^{L\times C}$ and its corresponding ground-truth label y∈{1,…,K}, the model predicts a probability distribution $\hat{y}\in R^{K}$ via a softmax classifier. The model parameters are optimized by minimizing the categorical cross-entropy loss:

(11) $$L_{\mathrm{CE}}=-\sum_{k=1}^{K}y_{k}\log\left({\hat{y}}_{k}\right),$$

where K=6 corresponds to the ISO 8608 road classes (A–F), $y_{k}$ is the one-hot encoded target label, and ${\hat{y}}_{k}$ is the predicted probability for class *k*.

## 4. Experiments

### 4.1. Dataset Construction and Preprocessing

To evaluate the performance of the proposed method in a controlled yet realistic environment, we constructed a high-fidelity vehicle vibration dataset. To ensure the representativeness of the data regarding real-world operating conditions, the road elevation profiles were synthesized strictly following the ISO 8608 international standard for road roughness classification. This standardization guarantees that the statistical properties of the generated profiles align with physical road surfaces found in engineering practice.

Data Generation. As detailed in Section 3, the road profiles for classes A–F were generated based on their power spectral density (PSD) characteristics. These profiles were then processed through a high-fidelity vehicle dynamics model in CarSim software (version 2021.0). Unlike simplified quarter-car models, this simulation environment captures the complex non-linear dynamic interactions between the tire, suspension, and vehicle body. The simulation collected vertical acceleration signals at a sampling rate of 1000 Hz for a duration of 300 s per road class, ensuring sufficient temporal diversity to cover stochastic road features.

Data Splitting and Preprocessing. To generate input samples for the deep learning model, a non-overlapping sliding window of 1 s (L=1000) was applied to the continuous time-series data. Consequently, the standard dataset comprises 1800 samples (300 samples × 6 classes). The dataset was randomly partitioned into training (70%), validation (20%), and testing (10%) sets.

Auxiliary Robustness Test Sets. In addition to the standard dataset collected under nominal conditions, we generated distinct auxiliary test sets to evaluate the model’s generalization capability against domain shifts. These sets include data under varying vehicle speeds (20 km/h), increased sprung mass (+480 kg), and environmental noise injection, which are specifically analyzed in Section 4.7.

### 4.2. Baselines and Metrics

We compare DB-MLP with a comprehensive set of baselines:

- RNN/CNN-based: LSTM, InceptionTime [25].
- Transformer-based: Transformer [15], Crossformer, Informer [16].
- MLP-based: TimeMixer [24], LightTS [23].

Evaluation metrics include Accuracy, Macro F1-Score, Parameter Count (Params), and Inference Time (averaged per sample).

### 4.3. Implementation Details

All models were implemented in PyTorch 1.7.1 on an NVIDIA RTX 4090 GPU.

- Training Protocol: We use the Adam optimizer ($lr=1\times 10^{-3}$) with an Early Stopping mechanism (patience = 60) to prevent overfitting.
- Batch Size: A batch size of 128 is used for MLP-based models, while it is adjusted to 32 for Transformer variants to accommodate GPU memory constraints.
- Unified Setting for Generalization: We adopted a unified protocol ($d_{\mathrm{model}}=128$, max epochs = 300) to ensure fair comparison without dataset-specific tuning.

### 4.4. Performance Evaluation

#### 4.4.1. Comparative Analysis with Baselines

Table 5 presents the quantitative comparison results. The proposed DB-MLP outperforms all baselines, achieving the highest accuracy (0.985) and F1-score (0.984).

Accuracy vs. Efficiency: While recent MLP-based models demonstrate strong performance, DB-MLP achieves a superior balance between accuracy and efficiency.

Specifically, compared to the second-best model TimeMixer (Acc. 0.965), DB-MLP improves accuracy by 2.0%. Remarkably, this gain is achieved with significantly lower resource consumption: DB-MLP requires only 6.7% of TimeMixer’s parameters (0.58 million vs. 8.55 million) and roughly 1% of its GPU memory (7.4 MB vs. 744.1 MB).

Compared to the lightweight baseline LightTS, DB-MLP not only improves accuracy by 3.0% (0.985 vs. 0.955) but also reduces inference latency by roughly a factor of three (0.007 ms vs. 0.024 ms). Overall, DB-MLP offers the fastest inference speed among all models, being approximately 20 times faster than InceptionTime and TimeMixer, making it the ideal candidate for real-time deployment on edge devices.

Architecture Analysis: The results highlight a clear trend: MLP-based architectures (TimeMixer, LightTS, DB-MLP) generally outperform RNN, CNN, and Transformer-based models in this task. This suggests that for fixed-length vehicle vibration signals, global temporal mixing is more effective than local convolution or recurrent processing. However, standard Attention-based models (e.g., Informer at 0.835) struggle significantly, likely due to the mismatch between sparse attention mechanisms and the need for capturing high-frequency fine-grained features. DB-MLP’s dual-branch design successfully overcomes these limitations by explicitly modeling both local details and global trends with linear complexity, leading to superior performance.

#### 4.4.2. Confusion Matrix Analysis

**Figure 6:**
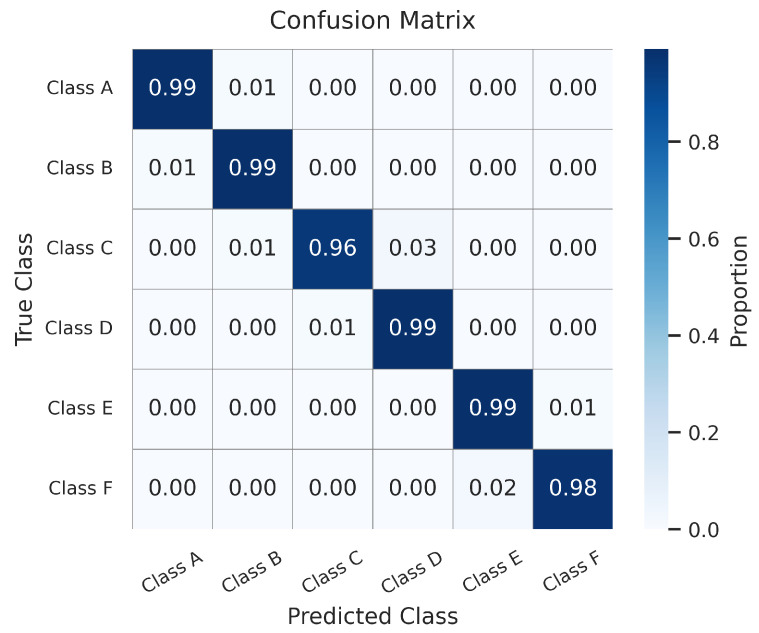
Confusion matrix of the proposed DB-MLP on the test set.

Figure 6 presents the confusion matrix derived from the testing set. The pronounced diagonal dominance corroborates the exceptional precision of the model. The limited number of misclassifications are strictly confined to adjacent categories, a pattern that reflects the inherent physical continuity of ISO 8608 road profiles. To effectively manage these boundary ambiguities in a real-world deployment setting, we propose a robust post-processing strategy. Given that the physical overlap between classes precludes absolute frame-level separation, the control logic necessitates the incorporation of a confidence margin defined by a minimum probability difference δ between the predicted and secondary classes. Furthermore, a temporal consistency check is employed to ensure that the minor stochastic fluctuations observed at class boundaries do not propagate instability to the downstream suspension control system.

### 4.5. Visual Analysis

**Figure 7:**
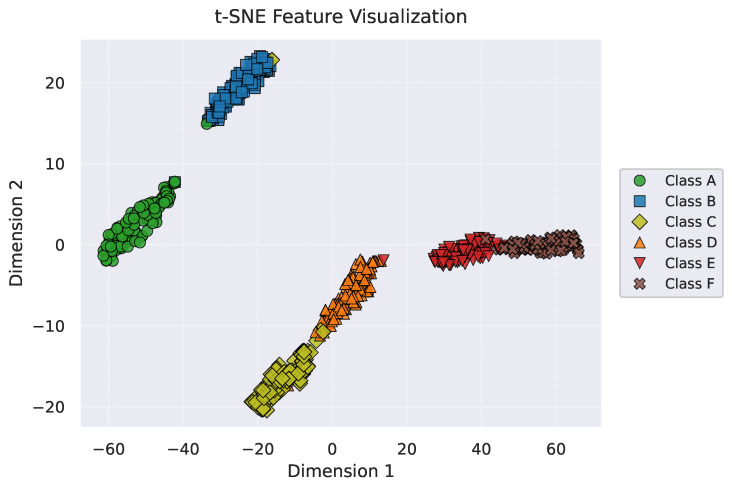
t-SNE feature visualization.

To interpret the learned representations, we visualize the feature manifold of the test set using t-SNE (Figure 7). The resulting topological structure is highly consistent with the vehicle dynamic responses illustrated in Figure 4. The model effectively distinguishes three physical regimes:

1. Tire-Filtering Stage (Classes A and B): The distinct isolation of these classes corresponds to the suspension-quiescent regime. On smooth roads, micro-texture excitations are primarily absorbed by tires, resulting in minimal sprung mass response. The model correctly clusters these as a distinct low-energy state.
2. Effective Stroke Regime (Classes C and D): As roughness increases, the suspension operates continuously within its effective design stroke. Although the damper exhibits non-linear force-velocity characteristics, the system dynamics remain smooth and bounded. The model maps Classes C and D as adjacent clusters with touching boundaries, accurately reflecting the continuous ordinal nature of the ISO 8608 spectrum before physical saturation occurs.
3. Saturation and Impact Point (Separation of D and E): A significant gap separates Class D from Class E. This structural discontinuity aligns with the transition from continuous oscillation to mechanical saturation (e.g., bump stop engagement). As observed in the severe regime responses (Classes E–F in Figure 4), the acceleration signals exhibit spikes characteristic of hard non-linear impacts. The model successfully detects this dynamics shift, separating the high-impact group from the nominal effective stroke group.

### 4.6. Ablation Study

To systematically evaluate the contribution of each component in the proposed DB-MLP architecture, we conducted two groups of ablation experiments: (i) Module Importance Analysis, employing a subtractive strategy, and (ii) Multi-Branch Depth Analysis, investigating the optimal number of temporal scales. All models share the same training settings to ensure a fair comparison.

#### 4.6.1. Ablation Settings

Module Importance.

To quantify the contribution of specific components, we configure five variants based on the subtractive “drop-out” principle:

- M0 (DB-MLP Ours): The complete architecture with dual-branch inputs, Time-Mixing, and Position-wise Feature Transformation enabled.
- M1 (w/o Multi-Scale): Disables the multi-branch structure. The model processes only the single-scale original sequence (1×) while retaining the Time-Mixing and Position-wise modules.
- M2 (w/o Position-wise Trans.): Removes the Adaptive Scaling block. The model retains the dual-branch inputs and Time-Mixing MLP for feature extraction.
- M3 (w/o Time-Mixing): Removes the global Time-Mixing MLP. The model retains the dual-branch inputs but relies on the Position-wise Feature Transformation for feature processing.
- M4 (MLP Projection Head): Removes all feature extraction modules. This baseline consists solely of the final MLP projection head applied directly to the flattened raw input, serving as a lower-bound reference.

Multi-Branch Depth Analysis.

We investigate the impact of temporal scale depth by varying the number of down-sampling branches:

- L0 (2 Branches): Combines the original sequence (1×) and the 2× down-sampled sequence. This is the default setting (Ours).
- L1 (3 Branches): Adds a coarser 4× down-sampled branch to L0.
- L2 (4 Branches): Further adds an 8× down-sampled branch to L1.

Training Protocol.

Consistent with the main experiments, all ablation variants are trained with a learning rate of $1\times 10^{-3}$ and early stopping with a patience of 60 epochs.

#### 4.6.2. Results and Analysis

Impact of Key Modules.

Table 6 summarizes the results. The full DB-MLP model achieves the highest accuracy of 98.5%.

- Dominance of Time-Mixing: The most significant performance drop occurs when removing the Time-Mixing module (M3), with accuracy plummeting to 81.7% (Δ=−16.8%). This drastic degradation confirms that the model’s primary capability stems from modeling global temporal dependencies via the Time-Mixing MLP.
- Importance of Non-Linearity: Removing the Position-wise Transformation (M2) results in a 2.3% drop in accuracy. This indicates that the non-linear feature recalibration is essential for refining the features extracted by the temporal mixer.
- Benefit of Multi-Scale: Simplifying the model to a single scale (M1) leads to a 1.3% decrease in accuracy. The dual-branch structure provides consistent gains by capturing patterns at different resolutions.
- Superiority over Baseline: The standalone MLP Projection Head(M4) achieves only 71.7% accuracy. The DB-MLP architecture yields a massive improvement of +26.8%, validating that the proposed dual-domain feature extraction is far more effective than a direct linear projection.

Impact of Branching Depth.

Table 7 illustrates the effect of varying the number of temporal scales.

- Optimal Configuration (L0): The dual-branch structure achieves the peak accuracy of 98.5%.
- Feature Degradation in Deeper Branches (L1 and L2): Notably, adding a third branch (L1, 4× down-sampling) causes the accuracy to drop back to 97.2%, identical to the single-scale baseline (M1). This suggests that the aggressive down-sampling filters out critical high-frequency vibration components (10–50 Hz), producing over-smoothed features that cancel out the benefits of the 2× branch. Further increasing the depth (L2) exacerbates this information loss (95.8%).

#### 4.6.3. Discussion

The ablation study leads to three key conclusions:

1. Time-Mixing is the Core Driver: Global temporal modeling is the cornerstone of the proposed method. Removing the Time-Mixing module (M2) causes the most drastic performance drop (Δ=−16.8%), confirming that the model relies on capturing long-range dependencies rather than simple statistical patterns.
2. Shallow Multi-Scale achieves the Best Balance: Our experiments identify the dual-branch structure (L0) as the optimal balance. While adding one down-sampled branch improves robustness, further increasing the depth (L1, L2) degrades performance due to spectral information loss and over-smoothing, rendering deeper branches ineffective.
3. Efficiency of Structured Feature Extraction: The DB-MLP achieves superior accuracy (98.5%) with 0.58 million parameters. In contrast, the standalone MLP Projection Head (M4) fails to capture complex patterns (71.7%), demonstrating that the performance gain comes from the specific dual-domain architecture rather than the classification head itself.

### 4.7. Robustness and Generalization Analysis

To validate the reliability of the proposed DB-MLP for real-world deployment, this section evaluates the model’s performance under three severe domain shifts: environmental sensor noise causing signal degradation, changes in vehicle mass resulting in parameter variations, and variations in driving speed causing frequency shifts.

#### 4.7.1. Resilience to Environmental Sensor Noise

To verify the model’s immunity to real-world interference, we conducted a stress test by injecting Additive White Gaussian Noise into the standardized sensor data strictly during the inference phase. The model was evaluated at Signal-to-Noise Ratios ranging from 30 dB down to 10 dB. This broad spectrum approximates unstructured environmental disturbances ranging from inherent sensor fluctuations to high-intensity mechanical vibrations.

**Figure 8:**
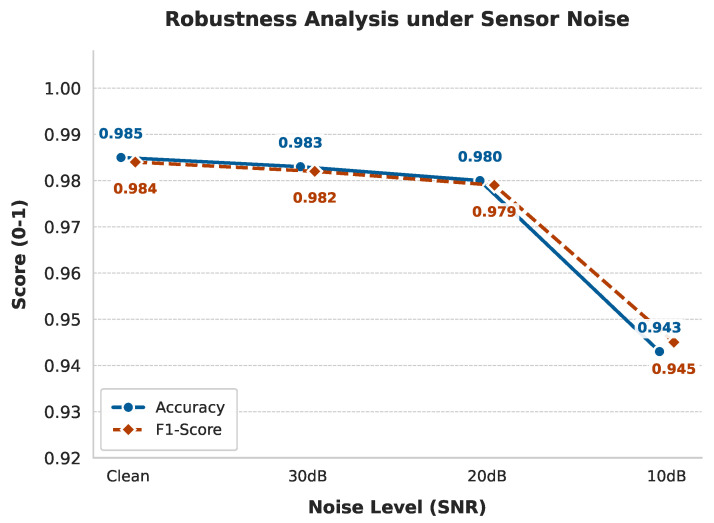
Performance curves of DB-MLP varying with sensor noise levels.

As illustrated in Figure 8, the results demonstrate strong robustness. Under typical noise conditions where SNR ≥ 20 dB, the accuracy at 20 dB showed a negligible drop compared to the clean baseline, indicating that standard sensor noise has virtually no effect. Even under severe interference at 10 dB, the model maintained a high accuracy of 94.3%. Notably, the F1-Score of 0.945 remained closely aligned with the accuracy, confirming balanced classification capabilities without bias.

We attribute this robustness to the dual-branch architecture, where the Average Pooling branch effectively functions as a low-pass filter, suppressing high-frequency noise while preserving essential road features.

#### 4.7.2. Adaptability to Vehicle Mass Variations

To further evaluate the model’s adaptability to vehicle parameter changes, a sensitivity test on the sprung mass was conducted. In real-world driving, passenger occupancy significantly alters the vehicle’s natural frequency according to $\omega_{n}=\sqrt{k/m}$, which can potentially affect signal patterns.

Experimental Setup.

Based on the simulation configuration, the baseline vehicle comprising only the driver has a total sprung mass of 2337 kg. A fully-loaded test set was generated by increasing the occupancy to 7 passengers, raising the total sprung mass to 2817 kg. This corresponds to a net mass increase of 480 kg, representing approximately a 20.5% variation. The model was trained solely on the baseline dataset and directly tested on this heavy-load dataset without fine-tuning.

Results.

Despite the significant shift in vehicle dynamics caused by the mass increase, the model maintained a high accuracy of 96.7%. This represents only a marginal decline of 1.8% compared to the baseline of 98.5%. This result confirms that the DB-MLP captures intrinsic road roughness features rather than overfitting to specific vehicle dynamic parameters.

#### 4.7.3. Generalization Across Driving Speeds via Physics-Guided Augmentation

Finally, this subsection validates the capability of the model to handle severe frequency shifts by testing on unseen 20 km/h data dominated by resonance using a model trained only on 36 km/h data dominated by isolation.

Motivation and Methodology.

Vehicle speed *v* linearly scales the signal frequency *f* according to $f=n_{0}\cdot v$. Consequently, lower speeds result in temporally stretched waveforms. Specifically, 20 km/h represents a worst-case scenario because it shifts road excitation frequencies into the vehicle’s resonance band (1–2 Hz). This creates high-amplitude body motion noise that makes classification significantly harder than at the nominal training speed of 36 km/h. To address this, we adopted a Single-Speed Training, Cross-Speed Testing protocol. A physics-guided resampling augmentation was applied to the 36 km/h training data to mathematically simulate the temporal patterns covering the range of 20–50 km/h, forcing the model to learn invariant spatial features $n_{0}$ rather than speed-dependent patterns.

Results and Quantitative Analysis.

As presented in Table 8, the frequency mismatch caused the Baseline (without augmentation) to drop to 84.4%. Our approach recovered performance to 90.0%, achieving an improvement of 5.6%. The remaining gap to the Oracle upper bound (95.3%) is expected and physically explainable: linear resampling cannot fully replicate the non-linear amplitude amplification caused by suspension resonance at specific frequencies.

**Figure 9:**
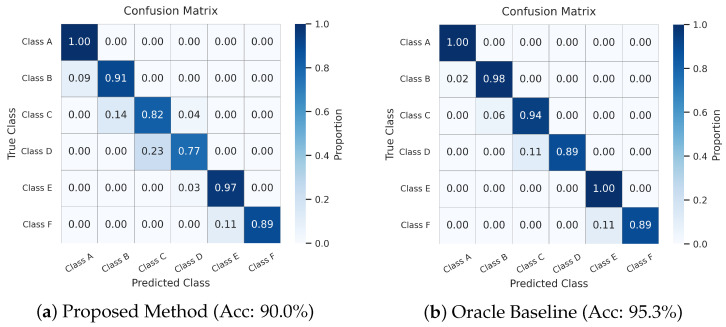
Confusion matrices on the 20 km/h test set. Note the structural similarity between (**a**,**b**), especially the identical performance on Class F.

Physical Consistency Analysis. The comparison of confusion matrices in Figure 9 reveals that the proposed method captures the true physical distribution of the target domain, despite the domain gap. Two key observations confirm this:

1. Resonance Amplitude Bias: We observed a slight tendency to underestimate roughness (e.g., Class D misclassified as C). This occurs because the mathematically resampled signals exhibit lower dynamic intensity than the actual resonating vehicle body dynamics at 20 km/h, leading to conservative predictions.
2. Identical Limit Behavior (Class F ≈ E): Remarkably, our accuracy on the most severe Class F roads is 0.89, which is identical to the Oracle baseline. Both models exhibit an 11% misclassification into Class E. This reflects a physical reality: at low speeds, the impact energy is insufficient to fully engage suspension stops, making Class F and E physically indistinguishable. This shared error pattern confirms the model has learned the genuine road dynamics rather than statistical artifacts.

#### 4.7.4. Summary and Discussion

In summary, the systematic evaluations in Section 4.7 demonstrate that DB-MLP maintains high precision across diverse domain shifts. Specifically, the model achieved 94.3% accuracy under environmental noise at 10 dB SNR, maintained 96.7% accuracy despite vehicle mass variations, and reached 90.0% accuracy under speed-induced frequency mismatches when utilizing PGA. These results confirm that the dual-branch architecture effectively captures intrinsic vibration features rather than over-fitting to specific vehicle parameters.

The 5.3% performance gap observed in the speed generalization test described in Section 4.7.3 warrants a deeper physical analysis. This discrepancy is primarily attributed to the frequency-selective gain of the suspension system. While linear resampling aligns the spectral components in the frequency domain, it does not account for the nonlinear magnitude amplification caused by resonance. Specifically, at 20 km/h, the excitation shifts into the primary resonance band of 1 to 2 Hz, where the system’s transmissibility |H(f)| is significantly higher than in the isolation zone at 36 km/h. To theoretically bridge this gap, a Magnitude Correction Factor Γ(f) can be defined to adjust the amplitude of resampled signals:

(12) $$\Gamma \left(f\right)=\frac{|H_{v_{target}}\left(f\right)|}{|H_{v_{source}}\left(f\right)|}$$

where the parameters are defined as follows:

- *f*: The vibration frequency component in Hz.
- |H(f)|: The magnitude of the suspension’s Frequency Response Function at frequency *f*.
- $v_{target}$ and $v_{source}$: The target driving speed of 20 km/h and the source training speed of 36 km/h, respectively.
- Γ(f): The frequency-dependent correction factor used to compensate for the gain difference between resonance and isolation states.

Regarding other engineering constraints, the model exhibits inherent structural immunity. Sensor bias, which manifests as a direct current offset, does not alter the alternating current patterns representing road roughness and is mitigated by our Global Normalization and Time-Mixing layers. Furthermore, minor sampling rate fluctuations, typically characterized as high-frequency jitter, are effectively smoothed by the Average Pooling branch which functions as a structural low-pass filter. Collectively, these design choices ensure that DB-MLP is not only computationally efficient but also robust against the non-ideal signal conditions typical of real-world vehicular environments. It is, however, essential to distinguish the physical operating domain of this data-driven approach from traditional geometric profiling. Since the proposed method fundamentally relies on dynamic vehicle-road interaction, intrinsic limitations exist under static conditions or negligible velocities where excitation is insufficient. Yet, this constraint is operationally acceptable for suspension control, as such systems predominately function during normal driving speeds where vehicle dynamics are significant.

## 5. Conclusions

This paper presents an effective and lightweight framework for road roughness classification using sprung mass vertical acceleration signals. By addressing the limitations of traditional linear simulation models and computationally intensive deep learning architectures, we achieved a favorable balance between classification accuracy and real-time inference efficiency.

First, we established a high-fidelity co-simulation platform combining CarSim and Simulink. Unlike simplified quarter-car models, this platform captures non-linear suspension dynamics and vehicle body coupling effects, generating a physically consistent dataset covering ISO A–F roughness levels. Second, we proposed DB-MLP, a novel dual-branch architecture tailored for vibration signal analysis. By employing multi-resolution temporal projection and dual-domain (time and position-wise) mixing, the model effectively extracts both fine-grained vibration textures and global motion trends without relying on heavy self-attention mechanisms. Crucially, this design explicitly decouples high-frequency road textures from low-frequency body motions, aligning feature extraction with physical vibration characteristics.

Experimental results demonstrate that DB-MLP achieves superior accuracy (98.5%) compared to established baselines on the specific road classification task. In terms of efficiency, it requires only 0.58 million parameters and achieves an ultra-low inference latency of 0.007 ms per sample, which is approximately 20 times faster than InceptionTime and TimeMixer. Such computational efficiency suggests that the model is highly suitable for deployment on resource-constrained embedded ECUs. Moreover, extensive robustness analyses validated the model’s stability under challenging conditions, including sensor noise, vehicle mass variations, and speed changes.

Future work will focus on two main directions: (1) Bridging the Sim-to-Real gap by validating the model on real-world vehicle test data and investigating transfer learning techniques to mitigate domain shifts caused by environmental noise and sensor heterogeneity; (2) Integrating the proposed classifier into closed-loop semi-active suspension control systems to evaluate its impact on improving not only ride comfort but also driving safety in extreme road conditions.

## Figures and Tables

**Table 1:** Six-level classification of road roughness.

Road Class	$G_{q}\left(n_{0}\right)/10^{-6}\;m^{3},\;n_{0}=0.1\;m^{-1}$		
	Lower Limit	Geometric Mean	Upper Limit
A	8	16	32
B	32	64	128
C	128	256	512
D	512	1024	2048
E	2048	4096	8192
F	8192	16,384	32,768

**Table 2:** Main vehicle and tire parameters used in the CarSim simulation.

Parameter (Unit)	Value
*Mass and Inertia*	
Sprung mass (kg)	2257
Unsprung mass, Front (kg)	139.4 (per side)
Unsprung mass, Rear (kg)	172.0
*Suspension and Tire*	
Suspension type (Front/Rear)	SLA/Hotchkiss
Spring stiffness, Front (N/mm)	189
Auxiliary roll stiffness, F/R (N·m/deg)	569/510
Damper configuration	Nonlinear (lookup table)
Tire simulation model	Internal Table Model
Tire size	265/70 R17
Vertical tire stiffness (N/mm)	440
Effective rolling radius (mm)	368
*Simulation Settings*	
Vehicle speed (km/h)	36
Sampling frequency (Hz)	1000

**Table 3:** Detailed architectural specifications of the DB-MLP.

Stage	Module	Configuration/Internal Operation	Output Shape
Input	Sprung Mass Acc (Normalized)	Sequence Length L=1000, Channel C=1	1000×1
1. Multi-Scale Inputs	Scale 1: Original	Identity Mapping	1000×1
	Scale 2: Downsampled	AvgPool (Window = 2, Stride = 2)	500×1
2. Dual-Branch MLP	Dual-Domain MLP Block	(Scale 1 Branch)	1000×1
	(Upper Path)	⌞ Time Mixing: Linear 1000→128→1000	
		⌞ Pos-wise Feature Trans.: Adaptive Scaling (1→1)	
	Dual-Domain MLP Block	(Scale 2 Branch)	500×1
	(Lower Path)	⌞ Time Mixing: Linear 500→128→500	
		⌞ Pos-wise Feature Trans.: Adaptive Scaling (1→1)	
3. Output Stage	Concatenation	Flatten and Concat (Scale 1 + Scale 2)	1500×1
	MLP Projection Head	Linear Projection (1500→128→6)	6
	Road Surface Grade	Softmax Probability Scores	6

**Table 4:** Summary of architectural design choices and their physical basis.

Architectural Component	Configuration	Physical Interpretation and Rationale
Branching Strategy	Dual-Branch at 1× and 2× scales	Spectral Decoupling: The 1× branch retains high-resolution information to characterize road textures (10–50 Hz), while the 2× branch provides a coarser temporal view, effectively isolating low-frequency sprung mass motions (<5 Hz).
Pooling Type	AvgPool Operation	Signal Smoothing: Functioning as a low-pass filter, AvgPool averages adjacent signals to suppress high-frequency noise while preserving the underlying energy trend, mitigating aliasing artifacts common in raw vibration data.
Down-sampling Rate	Stride set to 2	Frequency Focus: The reduced temporal resolution shifts the model’s focus from local micro-textures to global signal trends, naturally emphasizing the frequency range most relevant to passenger ride comfort.
Network Depth	Single Block per Branch	Parsimony Principle: Given that suspension dynamics are governed by low-dimensional physical laws, a shallow structure is sufficient to capture essential features. This design minimizes overfitting to stochastic noise and ensures ultra-low latency.

**Table 5:** Performance comparison on the simulated ISO 8608-based road roughness classification dataset.

Category	Model	Acc.	F1	Params (Million)	Infer Time (ms)	GPU Mem (MB)
RNN-based	LSTM	0.923	0.921	0.967	0.647	45.1
CNN-based	InceptionTime	0.925	0.924	0.919	0.144	70.9
Transformer	Transformer	0.922	0.920	1.490	0.561	1193.5
	Crossformer	0.925	0.921	2.350	0.179	28.2
	Informer	0.835	0.831	1.231	0.664	328.5
MLP-based	TimeMixer	0.965	0.964	8.552	0.139	744.1
	LightTS	0.955	0.954	1.122	0.024	12.4
	**DB-MLP (Ours)**	**0.985**	**0.984**	**0.579**	**0.007**	**7.4**

Note: Bold values indicate the best performance in each column.

**Table 6:** Module ablation results using a subtractive strategy. M4 represents the baseline using only the MLP Projection Head without feature extraction layers.

Model Variant	Params (Million)	Accuracy	F1-Score	Δ Acc
M0: DB-MLP (Ours)	0.579	0.985	0.984	-
M1: w/o Multi-Scale	0.386	0.972	0.971	↓ 1.3%
M2: w/o Pos-wise Trans.	0.579	0.962	0.960	↓ 2.3%
M3: w/o Time-Mixing	0.193	0.817	0.819	↓ 16.8%
M4: MLP Projection Head	0.129	0.717	0.708	↓ 26.8%

Note: The hyphen (-) denotes the reference baseline, and the downward arrow (↓) indicates a decrease in accuracy compared to M0.

**Table 7:** Impact of branching depth. Note that adding the 3rd branch (L1) degrades performance back to the single-scale level (M1), indicating spectral information loss.

ID	Configuration	Params (Million)	Accuracy	F1-Score
L0	2 Branches (Ours)	0.579	0.985	0.984
L1	3 Branches (+4×)	0.675	0.972	0.971
L2	4 Branches (+8×)	0.723	0.958	0.958

**Table 8:** Performance comparison on the 20 km/h resonance dataset.

Model	Training Data	Test Speed	Accuracy	Improvement
Baseline	36 km/h (Fixed)	20 km/h	84.4%	-
Ours	36 km/h (Augmented)	20 km/h	90.0%	+5.6%
Oracle	Real 20 km/h Data	20 km/h	95.3%	(Upper Bound)

Note: The hyphen (-) denotes the reference baseline.

## Data Availability

The data presented in this study are available on request from the corresponding author.

## Abbreviations

The following abbreviations are used in this manuscript:

DB-MLP	Dual-Branch Multi-Layer Perceptron
PSD	Power Spectral Density
ISO	International Organization for Standardization
MLP	Multi-Layer Perceptron
FFN	Feed-Forward Network
SLA	Short-Long Arm Suspension
ECU	Electronic Control Unit
t-SNE	t-Distributed Stochastic Neighbor Embedding
